# Body Mass Index and the Risk of Cardiovascular and All-Cause Mortality Among Patients With Hypertension: A Population-Based Prospective Cohort Study Among Adults in Beijing, China

**DOI:** 10.2188/jea.JE20150323

**Published:** 2016-12-05

**Authors:** Kuibao Li, Chonghua Yao, Xinchun Yang, Xuan Di, Na Li, Lei Dong, Li Xu, Meili Zheng

**Affiliations:** 1 Heart Center of Beijing Chaoyang Hospital Affiliated to Capital Medical University, Beijing, China; 2 Beijing Centers for Disease Control and Prevention, Anzhen Hospital Affiliated to Capital Medical University, Beijing, China; 3 Pharmacy Department, Beijing Chaoyang Hospital Affiliated to Capital Medical University, Beijing, China

**Keywords:** body mass index, death, cohort studies, hypertension, epidemiology

## Abstract

**Background:**

Studies on the association between body mass index (BMI) and death risk among patients with hypertension are limited, and the results are inconsistent. We investigated the association between BMI and cardiovascular disease (CVD) and all-cause mortality among hypertensive patients in a population of Beijing, China.

**Methods:**

We conducted a prospective cohort study of 2535 patients with hypertension aged 40 to 91 years from Beijing, China. Participants with a history of CVD at baseline were excluded from analysis. Cox proportional hazards regression models were used to estimate the association of different levels of BMI stratification with CVD and all-cause mortality.

**Results:**

During a mean follow-up of 8.1 years, 486 deaths were identified, including 233 cases of CVD death. The multivariable-adjusted hazards ratios for all-cause mortality associated with BMI levels (<20, 20–22, 22–24, 24–26 [reference group], 26–28, 28–30, and ≥30 kg/m^2^) were 2.03 (95% confidence interval [CI], 1.48–2.78), 1.61 (95% CI, 1.18–2.20), 1.30 (95% CI, 0.95–1.78), 1.00 (reference), 1.12 (95% CI, 0.77–1.64), 1.33 (95% CI, 0.90–1.95), and 1.66 (95% CI, 1.10–2.49), respectively. When stratified by age, sex, or smoking status, the U-shaped association was still present in each subgroup (*P* > 0.05 for all interactions). Regarding the association of BMI with CVD mortality, a U-shaped trend was also observed.

**Conclusions:**

The present study showed a U-shaped association of BMI with CVD and all-cause mortality among patients with hypertension. A lowest risk of all-cause mortality was found among hypertensive patients with BMI between 24 and 26 kg/m^2^.

## INTRODUCTION

Hypertension is considered to be the leading risk factor for cardiovascular mortality and accounts for a large proportion of premature deaths in China as well as in Western countries.^1^^,^^2^ Obesity is also recognized as a major global epidemic health problem and is associated with an increasing prevalence of hypertension.^3^^–^^5^ However, the relationship between body mass index (BMI) and mortality among patients with hypertension is unclear, and reports regarding their association are limited. Weight loss has been known to improve blood pressure in hypertensive patients.^6^ All major international guidelines recommend weight loss as part of the management of hypertension, but the BMI level associated with lowest mortality in these patients has not previously been well documented.^7^^,^^8^ Wang et al^9^ have reported that overweight and obesity were not associated with all-cause mortality in a group of Chinese elderly hypertensive patients. In one retrospective cohort study of adults with hypertension, a U-shaped relationship was observed between BMI and mortality.^10^ In contrast, a subgroup analysis of the Taiwan Longitudinal Study on Aging in patients with hypertension reported an inverse association between BMI levels and all-cause mortality.^11^ The reasons for the difference in associations across these studies are not clear; however, these studies differ in their design and sample sizes. The aim of the present study was to investigate the association of different levels of BMI at baseline with risk of all-cause and CVD mortality among patients with hypertension in a prospective cohort study conducted in Beijing, China.

## METHODS

### Study participants

In the 1991 China National Hypertension Survey, a multistage random cluster sampling design was used to select a representative sample of the general Chinese population aged 15 years or older from all 30 provinces in mainland China.^12^ In this investigation, the participants were responders to this National Hypertension Survey who were residents of Beijing, China. Of these, 7601 subjects who were ≥40 years old were scheduled to be followed up in 1996 and 1999 by trained staff. In this study, we excluded subjects with a history of coronary heart disease (myocardial infarction, angina pectoris, or revascularization) or stroke (*n* = 326) and those with no hypertension (*n* = 4321) at baseline. Hypertensive patients who could not be contacted during follow-up were also excluded (*n* = 395). Finally, we excluded patients with BMI in the most extreme 1% of the BMI distribution to minimize the effect of implausible values (*n* = 24). Thus, a total of 2535 patients with hypertension aged 40 to 91 years were left in this present study. Hypertensive patients who were excluded from the present study were comparable in terms of baseline characteristics with those included in this study, except for the former being younger (mean age, 57.6 versus 60.7 years).

This study was approved by the Tulane University Health Sciences Center Institutional Review Board and the Cardiovascular Institute and Fu Wai Hospital Ethics Committee. Written informed consent was obtained from all study participants involved in our study.

### Baseline examination

Trained staff interviewed participants using a standard questionnaire to obtain information on demographic characteristic, medical history, and lifestyle risk factors.^12^ The individuals were classified as smokers (current or former smokers) and non-smokers. “Current smokers” were defined as those who smoked at least 100 cigarettes in their lifetime and reported smoking every day or somedays in the 3 months prior to the baseline visit. “Former smokers” were defined as those who reported having smoked at least 100 cigarettes during their lifetime but had given up smoking for more than 3 months at the time of investigation. “Ever smokers” included both current and former smokers. “Never smokers” were respondents with a lifetime smoking history of fewer than 100 cigarettes and/or a report of “never smoking”. The cumulative amount of cigarette smoking was calculated as number of cigarettes smoked per day times years of smoking. Alcohol consumption was defined as drinking alcohol at least 12 times during the year prior to baseline investigation, and the rest were classified as non-drinkers. Body weight and height were measured using standard methods. BMI was calculated as weight in kilograms divided by the square of height in meters (kg/m^2^). Blood pressure and heart rate were measured after at least 10 minutes of rest in sitting position. Three blood pressure measurements were taken for each participant using a mercury sphygmomanometer according to a standard protocol. The mean of these three blood pressure measurements was used for data analysis. Resting heart rates were measured by palpating the radial pulse over a period of 15 seconds with a stop watch and multiplying by 4. If the pulse was irregular or difficult to count, the test was extended to 60 seconds, and a stethoscope was used at the apex of the heart and counted for a 60-second interval if necessary. Hypertension was defined as systolic blood pressure more than 140 mm Hg, diastolic blood pressure more than 90 mm Hg, or use of antihypertensive drugs. Control of hypertension was defined as systolic blood pressure less than 140 mm Hg and diastolic blood pressure less than 90 mm Hg after taking antihypertensive drugs.

### Follow-up and endpoints

The follow-up examinations, which were conducted in 1996 and 1999, included tracking study participants or their proxies to a current address, performing in-depth interviews to ascertain disease status and vital information, and obtaining hospital records and death certificates. The methods of outcome ascertainment were as follows: 1) all participants and families of the deceased or other witness of the fatal events were interviewed by trained staff; 2) if the invitees did not respond, the trained staff would visit the home or workplace of the participants to get the information on morbidity and vital status, including cause of death; 3) for those whose information could not be obtained using the aforementioned ways, we attempted to contact participants using telephone or mail; 4) all cases of death and the cause of death were ascertained by reviewing hospital charts or documents at local administrative offices, supplemented by interviews with local physicians, families, or witnesses of the fatal events. An end-point assessment committee reviewed and confirmed (or rejected) the hospital discharge diagnosis and cause of death based on the abstracted information using pre-established criteria.

The primary study outcomes for this study were deaths from all-causes and CVD. Causes of death were coded according to the International Classification of Diseases, Ninth Revision (ICD-9). The CVD deaths reported here were ICD-9 codes 390–459.

### Statistical analyses

When we explored the association between BMI and all-cause mortality, BMI was first evaluated in the following seven categories: <20, 20–22, 22–24, 24–26 (reference group), 26–28, 28–30, and ≥30 kg/m^2^. Due to the number of CVD deaths being relatively small in each BMI category, we combined some of these BMI categories and arrived at the following four BMI categories used to analyze the association between BMI and CVD mortality: <22, 22–26, 26–30, and ≥30 kg/m^2^. Continuous variables are shown as means (standard deviations). Categorical variables are shown as counts and percentages. Baseline characteristics were presented after age-adjustment using linear regression analyses for continuous variables and logit or ologit regression analyses for categorical variables. The association between BMI and CVD or all-cause mortality was analyzed using Cox proportional hazards models. All analyses were adjusted for baseline age and sex and further adjusted for smoking, alcohol drinking, and heart rate. Blood pressure was not adjusted in the primary analyses because it may be an intermediate factor in the causal path between BMI and mortality. However, the results were similar after further adjustment for this variable. In the final multivariable analysis, we used a cumulative amount of cigarette smoking instead of smoking status and added treatment and control situations of hypertension to further adjust for potential confounding.

We also modeled BMI as a continuous variable to assess whether a nonlinear association existed between BMI and the outcomes of interest. We used multivariable proportional hazards regression polynomial modeling of BMI to find a best fitting transformation of BMI by comparing model deviances using a χ^2^ distribution with 1 degree of freedom. Adjusted variables were the same as those in the final multivariable analysis modeling BMI as a categorical variable.

Subgroup analyses by sex, age (≤60 or >60 years), and cigarette smoking status were conducted. Effect modification by smoking status (never smokers vs ever or current smokers), age at diagnosis (≤60 or >60 years), and sex (male vs female) was estimated from the multiplicative interaction term between BMI categories and the effect modifier added to the main effects model.

To avoid the potential bias resulting from occult disease at baseline, additional analyses were conducted excluding the patients who died during the first 2 years of follow-up.

All analyses were performed using STATA 12 (Stata Corp, College Station, TX, USA), with two-tailed tests and a significance level of 0.05.

## RESULTS

Age-adjusted baseline characteristics of the study population are presented in Table 1. During a mean follow-up period of 8.1 years (20 643 person-years), 486 hypertensive subjects died from all causes, including 233 cases from CVD. All-cause and CVD mortality rates were 235 (95% confidence interval [CI], 215–257) and 113 (95% CI, 99–128) cases per 10 000 person-years, respectively.

**Table 1.** Age-adjusted baseline characteristics according to BMI categories among patients with hypertension

	BMI, kg/m^2^							

	<20	20–22	22–24	24–26	26–28	28–30	≥30	*P* value
*n*	263	373	492	514	398	278	217	
Mean (SD) age, years	66.8 (10.6)	62.3 (11.5)	60.9 (10.9)	59.5 (9.8)	58.3 (9.2)	59.3 (10.1)	58.9 (9.1)	<0.001
Male, %	43.4	44.0	44.2	44.4	44.5	44.4	44.5	<0.001
Mean (SD) systolic BP mm Hg	158 (6)	155 (7)	154 (6)	154 (6)	153 (5)	153 (6)	153 (5)	<0.001
Smoking status, %								<0.001
Never smokers	62.4	62.6	62.7	62.8	62.9	62.8	62.8	
Ever smokers	7.9	6.7	6.3	5.8	5.5	5.8	5.6	
Current smokers	29.7	30.7	31.0	31.3	31.6	31.4	31.6	
Mean (SD) cumulative number of cigarettes smoked	188 (20)	180 (22)	177 (20)	174 (18)	172 (17)	174 (19)	172 (17)	<0.001
Alcohol drinking, %	19.7	20.6	20.8	21.1	21.3	21.1	21.3	<0.001
Mean (SD) heart rate, beats/min	77 (0.1)	77 (0.1)	77 (0.1)	77 (0.1)	77 (0.1)	77 (0.1)	77 (0.1)	<0.001
Treatment proportions of HBP, %	25.8	28.5	29.3	30.1	30.8	30.3	30.7	<0.001
Control proportions of HBP, %	5.9	7.8	8.3	8.6	9.1	8.8	9.0	<0.001

BMI, body mass index; BP, blood pressure; HBP, high blood pressure; SD, standard deviation.

Data are presented as mean (SD) or percentage.

Risk of all-cause mortality was significantly increased among hypertensive subjects with baseline BMI <22 and ≥30 kg/m^2^ compared with subjects with baseline BMI of 24–26 kg/m^2^. After further adjustment for age, sex, cumulative amount of cigarette smoking, alcohol drinking, systolic blood pressure, treatment of hypertension, control of hypertension, and heart rate, this U-shaped association did not change among hypertensive subjects (Table 2).

**Table 2.** Hazard ratio of all-cause mortality according to different levels of BMI among patients with hypertension

	BMI, kg/m^2^						

	<20	20–22	22–24	24–26^c^	26–28	28–30	≥30
*n*	263	373	492	514	398	278	217
Deaths (%)	102 (38.8)	99 (26.5)	93 (18.9)	68 (13.2)	46 (11.6)	42 (15.1)	36 (16.6)
Person-years	1894.8	2909.4	3993.9	4336.3	3387.5	2321.4	1800.1
Mortality/10 000 person-y	538	340	233	157	136	181	200
Age- and sex-adjusted HR (95% CI)	2.09 (1.52–2.86)	1.68 (1.23–2.29)	1.31 (0.96–1.79)	1.00	1.00 (0.69–1.46)	1.21 (0.83–1.78)	1.65 (1.10–2.48)
Multivariable adjusted HR (95% CI)^a^	1.94 (1.42–2.67)	1.63 (1.19–2.23)	1.25 (0.91–1.71)	1.00	1.01 (0.69–1.46)	1.23 (0.84–1.81)	1.64 (1.09–2.47)
Multivariable adjusted HR (95% CI)^b^	2.03 (1.48–2.78)	1.61 (1.18–2.20)	1.30 (0.95–1.78)	1.00	1.12 (0.77–1.64)	1.33 (0.90–1.95)	1.66 (1.10–2.49)

BMI, body mass index; CI, confidence interval; HR, hazard ratio.

^a^Adjusted for age, sex, smoking, alcohol drinking and heart rate.

^b^Adjusted for age, sex, cumulative number of cigarette smoking instead of smoking status, alcohol drinking, heart rate, treatment of hypertension, control of hypertension, and systolic blood pressure.

^c^Reference group.

We observed a trend toward increased risk of CVD mortality among hypertensive subjects with BMI <26 and ≥30 kg/m^2^ compared with subjects with BMI of 26–30 kg/m^2^ (Table 3).

**Table 3.** Hazard ratio of CVD mortality according to different levels of BMI among patients with hypertension

	BMI (kg/m^2^)			

	<22	22–26	26–30^c^	≥30
*n*	636	1006	676	217
CVD deaths (%)	83 (10.1)	92 (9.2)	40 (5.9)	18 (8.3)
Person-years	4804	8330	5709	1800
Mortality/10 000 person-years	173	110	70	100
Age-and sex-adjusted HR (95% CI)	1.54 (1.05–2.27)	1.33 (0.92–1.93)	1.00	1.67 (0.95–2.91)
Multivariable adjusted HR (95% CI)^a^	1.43 (0.97–2.11)	1.30 (0.89–1.89)	1.00	1.61 (0.92–2.81)
Multivariable adjusted HR (95% CI)^b^	1.39 (0.94–2.06)	1.24 (0.85–1.80)	1.00	1.48 (0.85–2.60)

BMI, body mass index; CI, confidence interval; HR, hazard ratio.

^a^Adjusted for age, sex, smoking, alcohol drinking and heart rate.

^b^Adjusted for age, sex, cumulative number of cigarette smoking instead of smoking status, alcohol drinking, heart rate, treatment of hypertension, control of hypertension, and systolic blood pressure.

^c^Reference group.

When BMI was considered as a continuous variable, we found that a fractional polynomial regression model of second-degree (FP2) significantly improved a linear model regarding the relationship between BMI and all-cause mortality (*P* = 0.027), indicating a U-shaped association of BMI with all-cause mortality, with a nadir of the U-shape at BMIs of 24 to 26 kg/m^2^ (Figure 1). As for the relationship between BMI and CVD mortality, neither fractional polynomial regression model of second-degree (FP2) nor the model of first-degree (FP1) significantly improve a linear model (both *P* > 0.05); however, a trend toward a U-shaped association existed between BMI and CVD mortality (Figure 2).

**Figure.**
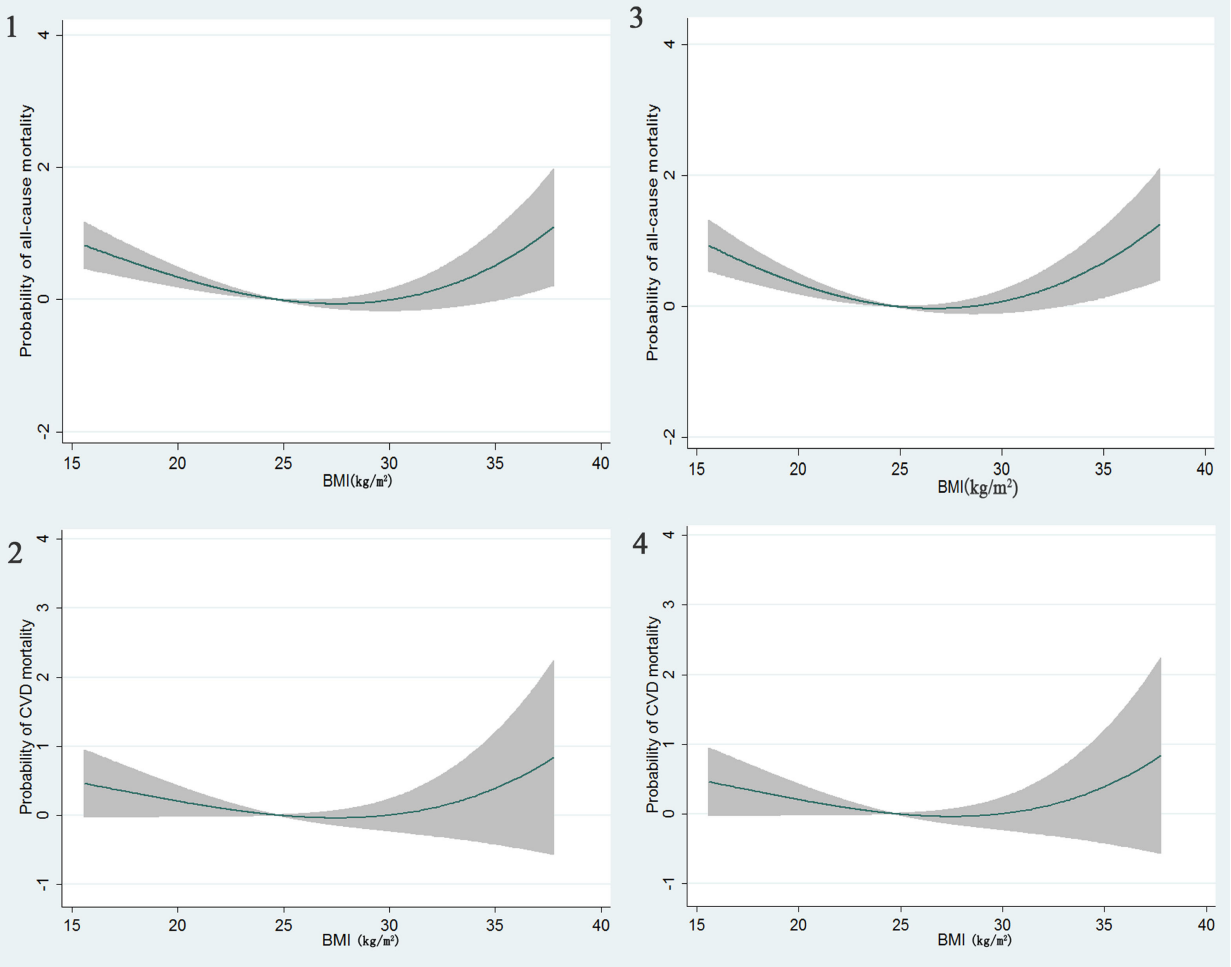
Predicted all-cause and CVD mortality with 95% confidence intervals based on the best fitting fractional polynomial models in the total participants (Figure 1: all-cause mortality; Figure 2: CVD mortality) and after excluding those who were censored during the first 2 years of follow-up (Figure 3: all-cause mortality; Figure 4: CVD mortality).

When we stratified the hypertensive population by age, sex, and smoking status, the associations between BMI and all-cause or CVD mortality were similar among the stratified subgroups, with the U-shaped associations being present in these subgroups (Table 4 and Table 5). No significant effect modification according to age, sex, or smoking status was observed in the present study (*P* > 0.05 for all interactions).

**Table 4.** Hazard ratio of all-cause mortality according to different levels of BMI among patients with hypertension of various subpopulations

	Person-years	Deaths, *n*	BMI, kg/m^2^							*P*^b^

			<20	20–22	22–24	24–26^a^	26–28	28–30	≥30	
Sex										0.614
Male	8991.2	246	2.56 (1.64–2.99)	1.90 (1.23–2.92)	1.38 (0.89–2.14)	1.00	1.10 (0.64–1.89)	1.50 (0.87–2.58)	1.83 (0.99–3.38)	
Female	11 652.2	240	1.55 (0.99–2.44)	1.36 (0.86–2.14)	1.22 (0.77–1.93)	1.00	1.16 (0.68–1.96)	1.18 (0.69–2.04)	1.48 (0.85–2.56)	
Age groups, years										0.256
≤60	10 923.7	79	3.05 (1.28–7.25)	2.50 (1.18–5.31)	1.54 (0.72–3.30)	1.00	1.10 (0.48–2.55)	1.55 (0.65–3.71)	1.44 (0.58–3.57)	
>60	9719.7	407	1.91 (1.36–2.69)	1.48 (1.05–2.08)	1.23 (0.87–1.74)	1.00	1.14 (0.75–1.74)	1.28 (0.83–1.96)	1.74 (1.10–2.76)	
Smoking status										
Never	13 091.0	269	1.97 (1.27–3.05)	1.65 (1.09–2.50)	1.46 (0.96–2.23)	1.00	1.22 (0.75–1.97)	1.27 (0.77–2.08)	1.69 (1.01–2.83)	0.663
Ever or Current	7552.4	217	1.92 (1.20–3.08)	1.50 (0.93–2.41)	1.06 (0.66–1.72)	1.00	0.98 (0.53–1.81)	1.47 (0.79–2.72)	1.56 (0.79–3.07)	

BMI, body mass index; CI, confidence interval; HR, hazard ratio.

Adjusted for age, sex, cumulative amount of cigarette smoking, alcohol drinking, systolic blood pressure, treatment of hypertension, control of hypertension, and heart rate.

^a^Reference group.

^b^*P* for interaction.

**Table 5.** Hazard ratio of CVD mortality according to different levels of BMI among patients with hypertension of various subpopulations

	Person-y	Deaths, *n*	BMI (kg/m^2^)				*P* for interaction

			<22	22–26	26–30^a^	≥30	
Sex							0.051
Male	8991.2	118	1.66 (0.92–2.95)	1.56 (0.90–2.70)	1.00	1.62 (0.67–2.94)	
Female	11 652.2	115	1.18 (0.68–2.04)	0.97 (0.57–1.64)	1.00	1.32 (0.64–2.72)	
Age groups, years							0.399
≤60	10 923.7	41	1.89 (0.80–4.51)	1.04 (0.47–2.33)	1.00	1.31 (0.44–3.91)	
>60	9719.7	192	1.36 (0.87–2.12)	1.27 (0.83–1.95)	1.00	1.55 (0.80–2.98)	
Smoking status							0.143
Never	13 091.0	132	1.28 (0.77–2.15)	1.28 (0.80–2.06)	1.00	1.62 (0.83–3.18)	
Ever or Current	7552.4	101	1.42 (0.76–2.67)	1.16 (0.63–2.16)	1.00	1.24 (0.45–3.45)	

BMI, body mass index; CI, confidence interval; HR, hazard ratio.

Adjusted for age, sex, cumulative number of cigarette smoking, alcohol drinking, heart rate, treatment of hypertension, control of hypertension, and systolic blood pressure.

^a^Reference group.

After the exclusion of individuals who died during the first 2 years of follow-up (*n* = 50), the multivariable-adjusted U-shaped relationship between BMI and all-cause or CVD mortality did not change (Figure 3 and Figure 4).

## DISCUSSION

In this community-based prospective cohort study in Beijing, the capital of China, we found a U-shaped association of BMI with all-cause mortality risk among patients with hypertension. A significantly increased risk of all-cause mortality was observed among patients with baseline BMI <22 and ≥30 kg/m^2^ compared with patients with baseline BMI of 24–26 kg/m^2^. Regarding the association of BMI with CVD mortality, a trend toward a U-shaped association also appeared to exist.

Limited studies have evaluated the association between BMI and total or CVD mortality among patients with hypertension, and results have been inconsistent. In the Minhang Community Study^9^ from China, Wang et al obtained information of 10 957 elderly hypertensive patients from an electronic health record system and followed them for a median of 3.7 years. They found that underweight, rather than overweight and obesity, was associated with all-cause mortality. In the Taiwan Longitudinal Study on Aging,^11^ which was conducted from 1996 to 2007, a subgroup analysis of middle-aged and older patients with hypertension (*n* = 985) found an inverse association between BMI and all-cause mortality. In contrast, the recently published results from one retrospective cohort study^10^ of 388 724 British adults aged 18 years or older with hypertension reported a U-shaped relationship between BMI and mortality in these patients over a median follow-up period of 8.0 years. In multivariable analysis, the lowest mortality was observed in participants with BMIs between 23.0 and 26.9 kg/m^2^. This result is consistent with the present study, in which we observed the lowest mortality among patients with BMI of 24 to 26 kg/m^2^. Furthermore, this U-shaped association was present in different age, sex, and smoking groups.

Several explanations may account for the apparent discrepancies in the association between BMI and mortality across studies, including differences in sample size, follow-up period, and study design. Obesity has been shown to lead to several diseases, including CVD. Death from these obesity-related diseases generally occurs long after disease onset. This could means a large sample size and long follow-up period are required to observe an association between higher BMI and death. In the Minhang Community Study, hypertensive patients were only followed for 3.7 years. While participants in the Taiwan Longitudinal Study were followed for nearly 10 years, only 985 hypertensives were included in the analysis. These issues with short follow-up and small sample size maybe the main reasons why these previous studies did not observe an association of higher BMI with all-cause mortality. In contrast, both our study and the study of British adults with hypertension possessed relatively larger sample sizes and longer follow-up periods and found a similar U-shaped association of BMI and mortality.

Age has been proposed as an effected modifier of the association between BMI and all-cause mortality in several studies.^13^^–^^15^ However, few studies have investigated the effect modification by age on the relationship between BMI and all-cause mortality among hypertensive patients. Our study showed that the U-shaped association between BMI and all-cause mortality was consistently present for hypertensive patients aged 40 to 60 years and 60 years or older. No significant effect modification according to age was identified.

Given that cigarette smoking has been shown to be associated with both low BMI and high mortality,^16^^,^^17^ and since subgroup analysis among persons who have never smoked can reduce residual bias related to smoking,^16^^,^^18^^–^^21^ subgroup analysis based on smoking status was conducted in the present study. Results showed that the U-shaped association between BMI and all-cause mortality was similar among hypertensive patients who never smoked and those who did smoke. We observed no significant effect modification according to smoking status.

Another major concern in the assessment of the association between BMI and mortality is the possibility of reverse causality. People with a history of cardiovascular disease or occult disease frequently lose weight; thus, people with a lower weight might have increased risk of death. To avoid such potential reverse causality, we excluded patients with a history of cardiovascular disease at baseline, and a sensitivity analyses was conducted by excluding deaths that occurred during the first 2 years of follow-up. The U-shaped association between BMI and all-cause mortality still existed in this sensitivity analysis.

We only observed a trend toward a U-shaped association of BMI with CVD mortality, in contrast with the significant non-linear association of BMI with all-cause mortality. The reason for the lack of statistical significance could be attributable to the relatively small number of CVD deaths.

Two strengths in our study warrant mention. First, in the present study, participants were randomly selected and enrolled from Beijing communities, with a participation rate of 93.6%, providing a highly generalizable community-based sample of middle-aged and older patients with hypertension. Second, apart from using BMI categories to explore the relationship between BMI and mortality in our analysis, we also used fractional polynomial regression to characterize the association, which can avoid the use of abrupt BMI cutoffs. The results from evaluating BMI using the aforementioned two ways were consistent in the present study.

There are also a few limitations in our investigation worth mentioning. First, although we excluded cardiovascular disease at baseline, we did not remove other potential diseases, which may confound the association between BMI and all-cause mortality. To eliminate the potential influence of preclinical disease, we excluded those who died during first 2 years and found that the results were similar to those derived from the whole cohort population. Second, the sample size in the present study was not larger enough to find significant differences in terms of all-cause mortality between some BMI categories and the reference category. Accordingly, we could not estimate an optimal BMI level in patients with hypertension from the present study. Third, we only used baseline BMI levels, and no repeat BMI levels were provided during follow-up, which may have introduced bias from a single baseline measurement. Fourth, 395 patients with hypertension could not be contacted during follow-up and were excluded from the present study. These patients were younger than the patients with hypertension included in the present study. Excluding these patients might have introduced selection bias. Fifth, we have no data regarding the differences of disease characteristics and lifestyles between the 1990s and now. To some extent, this may limit the possibility to extrapolate the results to current practice. However, we focused on the association between BMI and all-cause deaths in the present study, so differences in disease characteristics and lifestyles may have less effect on the association. Finally, it has been reported that exercise capacity has a modifier effect on the relationship between BMI and mortality risk in male veterans with hypertension.^22^ However, we did not measure this variable in the present study. Thus, the potential influence of unmeasured confounders, such physical activity and dietary factors, cannot be excluded.

Our study demonstrated a U-shaped association of BMI with all-cause or CVD mortality risk among middle-aged and older patients with hypertension. An increased risk of all-cause mortality was observed among patients with BMI <22 and ≥30 kg/m^2^ compared with patients with BMI between 24 and 26 kg/m^2^.
